# Assessing the Care Modality Preferences and Predictors for Digital Mental Health Treatment Seekers in a Technology-Enabled Stepped Care Delivery System: Cross-sectional Study

**DOI:** 10.2196/30162

**Published:** 2021-09-15

**Authors:** Elissa Kozlov, Meghan McDarby, Maximo Prescott, Myra Altman

**Affiliations:** 1 Institute for Health, Health Policy and Aging Research School of Public Health Rutgers University New Brunswick, NJ United States; 2 Department of Psychological and Brain Sciences Washington University in St. Louis St. Louis, MO United States; 3 Modern Health San Francisco, CA United States; 4 Clinical Excellence Research Center Stanford University Stanford, CA United States

**Keywords:** stepped care, technology, mental health care, patient-centered care

## Abstract

**Background:**

Access to mental health services continues to be a systemic problem in the United States and around the world owing to a variety of barriers including the limited availability of skilled providers and lack of mental health literacy among patients. Individuals seeking mental health treatment may not be aware of the multiple modalities of digital mental health care available to address their problems (eg, self-guided and group modalities, or one-to-one care with a provider). In fact, one-to-one, in-person treatment is the dominant care model with a masters- or doctoral-level trained mental health provider, and it may or may not be the appropriate or preferred level of care for an individual. Technology-enabled mental health platforms may be one way to improve access to mental health care by offering stepped care, but more research is needed to understand the care modality preferences of digital mental health care seekers because additional modalities become increasingly validated as effective treatment options.

**Objective:**

The purpose of this study was to describe and evaluate the predictors of care modality preferences among individuals enrolled in a technology-enabled stepped mental health care platform.

**Methods:**

This exploratory, cross-sectional study used employee data from the 2021 Modern Health database, an employer-sponsored mental health benefit that uses a technology-enabled platform to optimize digital mental health care delivery. Chi-square tests and one-way analysis of variance (ANOVA) were conducted to evaluate associations among the categorical and continuous factors of interest and the preferred care modality. Bivariate logistic regression models were constructed to estimate the odds ratios (ORs) of preferring a one-on-one versus self-guided group, or no preference for digital mental health care modalities.

**Results:**

Data were analyzed for 3661 employees. The most common modality preference was one-on-one care (1613/3661, 44.06%). Approximately one-fourth of the digital mental health care seekers (881/3661, 24.06%) expressed a preference for pursuing self-guided care, and others (294/3661, 8.03%) expressed a preference for group care. The ORs indicated that individuals aged 45 years and above were significantly more likely to express a preference for self-guided care compared to individuals aged between 18 and 24 years (OR 2.47, 95% CI 1.70-3.59; *P*<.001). Individuals screening positive for anxiety (OR 0.73, 95% CI 0.62-0.86; *P*<.001) or depression (OR 0.79, 95% CI 0.66-0.95; *P*=.02) were more likely to prefer one-on-one care.

**Conclusions:**

Our findings elucidated that care modality preferences vary and are related to clinical severity factors and demographic variables among individuals seeking digital mental health care.

## Introduction

Equitable access to mental health services continues to be a systemic problem in the United States and around the world [1]. Barriers to treatment for mental disorders include attitudinal barriers (eg, treatment skepticism) and structural barriers (eg, insufficient mental health workforce) [2]. An especially potent structural barrier to accessing mental health services is that prospective patients have challenges identifying and accessing viable treatment options [3], a key element of mental health literacy [4]. Importantly, individuals seeking mental health treatment may not understand the range of options available to address their problem, let alone expressing preferences for different modalities of receiving digital mental health care (eg, self-guided or group care, and one-to-one care with a provider). In fact, the dominant care model of one-to-one, in-person treatment involving a masters- or doctoral-level trained mental health provider may or may not be the appropriate or preferred level of care for an individual. This model of care, which requires access to trained and often expensive mental health specialists, partially explains the worldwide treatment gap in mental health care, as only a fraction of individuals with mental health needs receive treatment [5,6]. As a result, health care delivery systems have attempted to develop solutions that increase patient access to a variety of care options and account for barriers to treatment such as low mental health literacy and provider shortages. These models are known as “stepped care” approaches, which attempt to match patients to care options based on symptom severity and perceived needs [7,8].

Although they are not regularly available to the general population, technology-enabled mental health platforms may be one way to improve access to mental health care [9]. These platforms have the potential to streamline and optimize mental health care by matching patients’ presenting problems, severity, and treatment modality preferences. Importantly, these platforms have the potential to create an opportunity for individuals to access the treatment modality that matches their primary mental health concern while simultaneously improving mental health literacy and removing a structural barrier. These platforms allow patients to enter key demographic information, complete clinical assessments, describe their preferred areas of treatment focus, and express preferences for treatment modalities. The platforms then deploy an algorithm that accommodates and synthesizes this information, and patients are “matched” with a treatment approach and modality that considers their concerns and severity needs. Although these platforms have the potential to improve mental health outcomes, they are not regularly integrated into the existing mental health infrastructure.

Additionally, technology-enabled mental health platforms have the potential to optimize access to mental health services by facilitating stepped care in digital mental health treatments. Although there are many people seeking mental health care, some care seekers may not need or want traditional one-on-one psychotherapy given their presenting problem and severity level. A recent study that assessed care modality preferences found that less than half (44.5%) of patients with depression preferred in-person psychotherapy over digital mental health treatments (self-, peer- or provider-guided treatment) [10]. The stepped care approach posits that many care seekers would benefit from less resource-intensive treatments such as self-guided or group-based digital mental health treatments, which are more scalable than individual, in-person psychotherapy treatments. For certain populations with subclinical symptoms or areas of concern outside of traditional psychopathology, there may be no supporting evidence or need for individual psychotherapy from expensive and difficult-to-find specialists.

As more evidence-based modalities of receiving digital mental health care emerge— including self-guided interventions delivered via the internet or mobile health (mHealth) technology [11,12], group-based videoconferencing [13], and video-delivered individual psychotherapy sessions with a provider [14]—it is essential to better describe and understand the predictors of patient preferences for these modalities of digital mental health care. Prior research has demonstrated that individuals express preferences for mental health care when asked, and when those preferences are not met, the psychological outcomes are affected [15]. Existing clinical guidelines also encourage providers to incorporate patient preferences when evaluating treatment options wherever possible [16]. To facilitate patient-centered stepped care, more research is needed to understand care modality preferences because additional digital mental health treatments are becoming increasingly validated as effective options.

The purpose of this exploratory cross-sectional analysis of existing data was to examine modality preferences among individuals seeking digital mental health treatments through a technology-enabled, stepped care platform. We analyzed data from employees who registered with Modern Health, an employer-sponsored mental health benefit that uses a technology-enabled platform to optimize mental health care delivery. Our aim was to describe the care modality preferences of digital mental health treatment seekers and evaluate the associations among demographic factors, clinical factors, and the primary reasons for seeking care. We hypothesized that digital mental health care seekers with demographic characteristics traditionally associated with fewer treatment-seeking behaviors, such as being older (40 years and above) and being males, would be more likely to state a preference for self-guided care rather than traditional one-on-one treatment. We also hypothesized that individuals with higher levels of clinical severity would be more likely to state a preference for one-on-one care.

## Methods

### Intervention

Modern Health utilizes a stepped care approach to mental health care by directing users to the appropriate level of care when initiating treatment. All users answer a series of questions during registration to determine if their care needs correspond to preventive care, moderate clinical care, or high clinical care. The platform assesses clinical needs as well as each user’s care modality preferences to tailor treatment recommendations. Given that the study period coincided with the COVID-19 pandemic, only digital mental health treatments were available to the users of the platform and included the following: self-guided digital courses, group support via videoconferencing, one-to-one telecoaching with in-app texting, and one-to-one teletherapy (video-delivered individual psychotherapy sessions) with a licensed mental health specialist. The self-guided digital courses include guided meditations and modules that cover topics such as cognitive behavioral therapy, stress management, resilience and coping, burnout, and establishing healthy habits.

### Participants

The participants were employees (N=3661) who registered to use a mental health benefits platform between February 18, 2021, and April 9, 2021, and had provided complete registration data. Because Modern Health gradually rolled out the registration assessment, participants with missing data do not reflect poor responses but rather differences in when the registration portal was updated for different users. We analyzed data from individuals who were 18 years or older, had access to a smartphone, tablet, or computer, completed all baseline assessment questions through the Modern Health platform, and had their demographic data recorded. This study was reviewed by the WIRB-Copernicus Group Institutional Review Board (WCG IRB) and determined to be exempt from Institutional Review Board oversight.

### Procedures

Eligible employees register for Modern Health using a mobile app or via a website. Upon registering, participants complete a baseline assessment that includes the World Health Organization-5 Well-being Index (WHO-5), Patient Health Questionnaire-2 (PHQ-2), Generalized Anxiety Disorder 2-item (GAD-2) questionnaire, and a questionnaire about their primary focus areas and their care modality preference.

### Measures

#### Demographics

Employers optionally provided the gender and age data for employees eligible to use the Modern Health benefit prior to registration.

#### Well-being

Well-being was assessed using the WHO-5, a robust and unidimensional assessment of subjective well-being that has high psychometric validity as well as adequate sensitivity and specificity to screen for depressive symptoms [17]. Scores range on the percentage scale from 0 to 100 with higher scores indicating greater well-being.

#### Depression

The PHQ-2 was used to screen for depression. The PHQ-2 asks individuals if they have been feeling down, depressed, or hopeless and if they have had little interest or pleasure in doing things. The score totals range from 0 to 6 and cutoff scores higher than 3 are considered a positive screen for depression. In a recent study of community-based participants, the PHQ-2 showed a sensitivity of .64 and specificity of .85, which were comparable to the longer version of the scale, the PHQ-9 [18].

#### Anxiety

The GAD-2 was used to screen for anxiety. The GAD-2 is a psychometrically robust screener for anxiety that asks participants if they have been feeling nervous, anxious, or on the edge and if they have had difficulties in being able to stop or control worrying. The total scores on the GAD-2 range from 0 to 6, with scores higher than 3 indicating a positive screen for a clinically significant anxiety disorder. In a recent study of community-based participants, the GAD-2 showed a sensitivity of .71 and a specificity of .69, which were comparable to the longer version of the scale, the GAD-7 [18].

#### Topic Selection

The topics that participants selected during onboarding as their reason for visiting the platform were organized by their corresponding well-being dimensions (“my emotions,” “my physical well-being,” “my relationships,” and “my finances”). They selected these from a pre-established list of potential topics, such as anxiety, depression or low mood, improving my relationships and communication, burnout, and general professional development. The participants could not enter their own topics; they had to choose from the pre-established list.

#### Functional Impairment

An item adapted from the WHO Short Disability Assessment was used to assess functional impairment. Participants were asked, “In the past 2 weeks, (topic selections) have made it difficult for me to function in my life at home and work.” The response options followed a Likert scale including “strongly agree,” “agree,” “neither disagree or agree,” “disagree,” and “strongly disagree.”

#### Care Modality Preferences

Care modality preferences were assessed for individuals seeking digital mental health treatments on the platform by asking, “When it comes to improving my mental health, I prefer to work:…” Participants were able to select a single answer from the following response options: “on my own (self-guided, at my own pace),” “with a small group (live community sessions led by care professionals),” “one-on-one (meet with a care professional),” or “I’m not sure.”

### Statistical Analysis

Data cleaning and analysis was performed using R (version 4.0.3), a statistical software. WHO-5 scores were mean-centered and scaled to improve interpretability during regression modeling, such that a value of 0 represents the mean and an increase of 1 unit represents a difference of 1 SD. A complete case analysis was performed such that an individual’s data were only included if registration was completed and demographic data were available. Descriptive statistics were used to describe the demographic, clinical, and primary reasons for seeking care, and care modality preference characteristics of the sample. Chi-square and Kruskal-Wallis tests were used to evaluate associations between the categorical and continuous factors of interest and the preferred care modality, respectively. Bivariate logistic regression models were constructed to estimate the odds ratios (ORs) describing the relative differences in the odds of selecting self-guided or group modalities or being unsure of modality preferences compared to the odds of a preference for one-on-one care within each factor of interest.

## Results

### Descriptive Data

The mean age of respondents was 35.2 years (SD 9.4; range 19-74). The sample comprised mostly females (2113/3661, 57.7%). Respondents reported mean well-being scores of 43.33 (IQR 28; range 0-100), which can be interpreted as reduced well-being according to a commonly used cutoff score of 50 [17]. The primary topic selection endorsed most frequently by respondents was “my emotions” (1772/3661, 48.4%), followed by “my professional life” (707/3661, 19.3%), “my relationships” (560/3661, 15.3%), “my physical well-being” (549/3661, 15%), and lastly, “my finances” (73/3661, 2%). Approximately 35% of the sample (1271/3661) screened positive for anxiety, and 22.4% of the respondents (819/3661) screened positive for depression. The most selected care modality preference was traditional one-on-one care (1613/3661, 44.06%). Approximately one-fourth of the respondents (881/3661, 24.06%) expressed a preference for obtaining self-guided care. Less than 10% of the respondents (294/3661, 8.03%) reported a preference for small-group care options, whereas nearly a quarter of the respondents (873/3661, 23.85%) were unsure of their preferred treatment modality. The demographic, clinical, and topic selection characteristics differed significantly across care modality preferences. Table 1 presents the descriptive data.

**Table 1:** Descriptive statistics and associations between preferred care modalities and demographic, clinical, and primary reasons for seeking care.

Factor			Care modality preference											*P* value
			Total (N=3661)		On my own (self-guided) (n=881, 24.06%)		One-on-one care (meet with a care professional) (n=1613, 44.06%)		With a small group (live community sessions led by care professionals) (n=294, 8.03%)		I'm not sure (n=873, 23.85%)			
**Age (years), n (%)**														<.001^a^
	18-24	243 (6.6)		58 (6.6)		119 (7.4)		15 (5.1)		51 (5.8)				
	25-34	1900 (51.9)		397 (45.1)		925 (57.3)		141 (48)		437 (50.1)				
	35-44	895 (24.4)		214 (24.3)		393 (24.4)		75 (25.5)		213 (24.4)				
	45+	623 (17)		212 (24.1)		176 (10.9)		63 (21.4)		172 (19.7)				
**Sex, n (%)**														<.001^a^
	Female	2113 (57.7)		443 (50.3)		955 (59.2)		181 (61.6)		534 (61.2)				
	Male	1548 (42.3)		438 (49.7)		658 (40.8)		113 (38.4)		339 (38.8)				
**Subjective well-being (WHO-5^b^ score)**														<.001^c^
	Mean	43.33		47.56		40.63		47.01		42.81				
	IQR	28		28		32		24		28				
	Median (range)	44 (0-100)		48 (0-100)		40 (0-100)		44 (0-92)		40 (0- 100)				
**PHQ-2^d^ screening result, n (%)**														<.001^a^
	Negative depression screen (score<3)	2842 (77.6)		762 (86.5)		1172 (72.7)		236 (80.3)		672 (77)				
	Positive depression screen (score ≥3)	819 (22.4)		119 (13.5)		441 (27.3)		58 (19.7)		201 (23)				
**GAD-2^e^ screen result, n (%)**														<.001^a^
	Negative anxiety screen (score <3)	2390 (65.3)		703 (79.8)		911 (56.5)		217 (73.8)		559 (64)				
	Positive anxiety screen (score >3)	1271 (34.7)		178 (20.2)		702 (43.5)		77 (26.2)		314 (36)				
**Functional impairment, n (%)**														<.001^a^
	Strongly agree	455 (12.4)		57 (6.5)		288 (17.9)		22 (7.5)		88 (10.1)				
	Agree	1536 (42)		308 (35)		726 (45)		121 (41.2)		381 (43.6)				
	Neither disagree nor agree	864 (23.6)		231 (26.2)		329 (20.4)		74 (25.2)		230 (26.3)				
	Disagree	579 (15.8)		200 (22.7)		200 (12.4)		54 (18.4)		125 (14.3)				
	Strongly disagree	227 (6.2)		85 (9.6)		70 (4.3)		23 (7.8)		49 (5.6)				
**Primary focus area, n (%)**														<.001^a^
	My emotions	1772 (48.4)		327 (37.1)		896 (55.5)		129 (43.9)		420 (48.1)				
	My finances	73 (2)		26 (3)		27 (1.7)		7 (2.4)		13 (1.5)				
	My physical well-being	549 (15)		242 (27.5)		104 (6.4)		59 (20.1)		144 (16.5)				
	My professional life	707 (19.3)		200 (22.7)		284 (17.6)		62 (21.1)		161 (18.4)				
	My relationships	560 (15.3)		86 (9.8)		302 (18.7)		37 (12.6)		135 (15.5)				

^a^Pearson chi-square test.

^b^WHO-5: World Health Organization-5 Well-being Index.

^c^Kruskal-Wallis test.

^d^PHQ-2: Patient Health Questionnaire-2.

^e^GAD-2: Generalized Anxiety Disorder-2.

### Associations Between Demographic Characteristics and Care Modality Preferences

The results of the multinomial logistic regression analysis are presented in Table 2. Preferring a self-guided care modality over one-on-one care with a provider was significantly associated with older age, being males, higher well-being, screening negative for anxiety or depression, and reporting less functional impairment. The ORs indicated that individuals aged 45 and above were significantly more likely to prefer self-guided care over one-on-one care compared to individuals aged between 18 and 24 (OR 2.47, 95% CI 1.70-3.59; *P*<.001). Respondents identifying themselves as males were also significantly more likely to prefer self-guided care (OR 1.43, 95% CI 1.22-1.69; *P*<.001). More reports of well-being predicted a preference for self-guided care (OR 1.45, 95% CI 1.34-1.58; *P*<.001). Individuals who screened positive for anxiety (OR 0.33, 95% CI 0.27-0.40; *P*<.001) or depression (OR 0.42, 95% CI 0.33-0.52; *P*<.001) were significantly less likely to prefer self-guided care. The likelihood of preferring self-guided care was significantly lower among individuals who neither agreed nor disagreed (OR 0.58, 95% CI 0.40-0.83; *P*=.003), agreed (OR 0.35, 95% CI 0.25-0.49; *P*<.001), or strongly agreed (OR 0.16, 95% CI 0.11-0.25; *P*<.001) that their topic selection caused functional impairment. In addition, individuals who selected “my finances” (OR 2.64, 95% CI 1.52-4.59; *P*=.001)*,* “my physical well-being” (OR 6.38, 95% CI 4.9-8.29; *P*<.001)*,* or “my professional life” (OR 1.93, 95% CI 1.55-2.41; *P*<.001) as their topic were significantly more likely to prefer a self-guided modality compared to individuals who reported “my emotions” as a primary area of focus.

A preference for a group care modality over one-on-one care with a provider was significantly associated with older age, higher well-being, screening negative for anxiety or depression, and reporting less functional impairment. Respondents aged 45 and above were significantly more likely to prefer group care (OR 2.84, 95% CI 1.54-5.22; *P*<.001). More reports of well-being also predicted a preference for group care (OR 1.41, 95% CI 1.24-1.60; *P*<.001). Individuals who screened positive for anxiety (OR 0.46, 95% CI 0.35-0.61; *P*<.001) or depression (OR 0.65, 95% CI 0.48-0.89; *P*=.007) were significantly less likely to prefer group care. The likelihood of preferring group care over one-on-one care was significantly lower among individuals who agreed (OR 0.51, 95% CI 0.30-0.84; *P*=.009) or strongly agreed (OR 0.23, 95% CI 0.12-0.44; *P*<.001) that their topic selection had resulted in functional impairment. Respondents who indicated that “my physical well-being” was their primary area of focus were significantly more likely to prefer a group care modality (OR 3.94, 95% CI 2.72-5.70; *P*<.001), as was the case for respondents who reported that “my professional life” was their primary area of focus (OR 1.52, 95% CI 1.09-2.11; *P*=.014).

Being unsure about one’s preference for treatment over one-on-one care with a provider was significantly associated with older age, greater well-being, screening negative for anxiety or depression, and reporting less functional impairment. Individuals aged over 45 years were more likely to be unsure about their treatment modality preference (OR 2.28, 95% CI 1.54-3.37; *P*<.001). Individuals reporting higher well-being were significantly more likely to be unsure about their treatment modality preference (OR 1.13, 95% CI 1.04-1.23; *P*=.005). Individuals who reported “my physical well-being” as their primary topic were significantly more likely to report that they were unsure about their treatment modality preferences (OR 2.95, 95% CI 2.24-3.90; *P*<.001).

Screening positive for anxiety (OR 0.73, 95% CI 0.62-0.86; *P*<.001) or depression (OR 0.79, 95% CI 0.66-0.95; *P*=.019) was significantly associated with a preference for one-on-one care. The likelihood of preferring one-on-one care was significantly higher among individuals who strongly agreed that their topic selection had caused functional impairment (OR 0.44, 95% CI 0.28-0.68; *P*<.001).

**Table 2:** Comparison of care modality preferences based on bivariate multinomial logistic regression results for relative associations between preferred care modalities and demographic, clinical, and primary reasons for seeking treatment.

Factor			Care modality preference distribution									Self-guided vs 1:1 with provider							Group vs 1:1 with provider							Unsure vs 1:1 with provider				
			1:1 (Ref^a^) (%)		Self-guided (%)		Group (%)		Unsure (%)		OR^b^			95% CI		*P* value		OR			95% CI		*P* value		OR			95% CI		*P* value
**Age (years)**																														
	18-24 (ref)	49		23.9		6.2		21		1			—^c^		—		—			—		—		—			—		—	
	25-34	48.7		20.9		7.4		23		0.88			0.63- 1.23		.46		1.21			0.69- 2.13		.50		1.1			0.78- 1.56		.58	
	35-44	43.9		23.9		8.4		23.8		1.12			0.78- 1.59		.54		1.51			0.84- 2.73		.17		1.26			0.88- 1.83		.21	
	45+	28.3		34		10.1		27.6		2.47			1.7- 3.59		<.001		2.84			1.54- 5.22		.001		2.28			1.54- 3.37		.001	
**Sex**																														
	Female (ref)	45.2		21		8.6		25.3		1			—		—		—			—		—		—			—		—	
	Male	42.5		28.3		7.3		21.9		1.43			1.22- 1.69		<.001		0.91			0.7- 1.17		.50		0.92			0.78- 1.09		.30	
**Subjective well**-**being**																														
	WHO-5 score^d^	—		—		—		—		1.45			1.34- 1.58		<.001		1.41			1.24-1.60		<.001		1.13			1.04-1.23		.005	
**Depression**																														
	Negative PHQ-2^e^ screen (ref)	41.2		26.8		8.3		23.6		1			—		—		—			—		—		—			—		—	
	Positive PHQ-2 screen	53.8		14.5		7.1		24.5		0.42			0.33- 0.52		<.001		0.65			0.48-0.89		.007		0.79			0.66-0.96		.02	
**Anxiety**																														
	Negative GAD-2^f^ screen (ref)	38.1		29.4		9.1		23.4		1			—		—		—			—		—		—			—		—	
	Positive GAD-2 screen	55.2		14		6.1		24.7		0.33			0.27- 0.4		<.001		0.46			0.35- 0.61		<.001		0.73			0.62-0.86		<.001	
**Functional impairment**																														
	Strongly agree	63.3		12.5		4.8		19.3		0.16			0.11- 0.25		<.001		0.23			0.12- 0.44		<.001		0.44			0.28-0.68		<.001	
	Agree	47.3		20.1		7.9		24.8		0.35			0.25- 0.49		<.001		0.51			0.3- 0.84		.009		0.75			0.51-1.1		.14	
	Neither agree nor disagree	38.1		26.7		8.6		26.6		0.58			0.4- 0.83		.003		0.68			0.4- 1.17		.17		1			0.67- 1.49		.99	
	Disagree	34.5		34.5		9.3		21.6		0.82			0.57- 1.19		.31		0.82			0.47-1.44		.50		0.89			0.58-1.37		.60	
	Strongly disagree (ref)	30.8		37.4		10.1		21.6		1			—		—		—			—		—		—			—		—	
**Primary focus area**																														
	My emotions (ref)	50.6		18.5		7.3		23.7		1			—		—		—			—		—		—			—		—	
	My finances	37		35.6		9.6		17.8		2.64			1.52- 4.59		.001		1.8			0.77-4.22		.18		1.03			0.52-2.01		.94	
	My physical well-being	18.9		44.1		10.7		26.2		6.38			4.9- 8.29		<.001		3.94			2.72-5.7		<.001		2.95			2.24-3.9		<.001	
	My professional life	40.2		28.3		8.8		22.8		1.93			1.55- 2.41		<.001		1.52			1.09-2.11		.014		1.21			0.97-1.52		.10	
	My relationships	53.9		15.4		6.6		24.1		0.78			0.6- 1.02		.07		0.85			0.58-1.25		.41		0.95			0.75- 1.2		.70	

^a^Ref: reference.

^b^OR: odds ratio.

^c^Not applicable.

^d^WHO-5: World Health Organization-5 Well-being Index. The scores are mean-centered and scaled to improve interpretability.

^e^PHQ-2: Patient Health Questionnaire-2.

^f^GAD-2: Generalized Anxiety Disorder-2.

## Discussion

### Principal Results

This study revealed that in a large sample of adults seeking digital mental health care with access to an employer-sponsored mental health benefit, fewer than half of the respondents indicated that they preferred one-to-one care. Nearly one-fourth of the respondents did not have a modality preference, and the remaining sample preferred self-guided care or group care, revealing substantial variability in care modality preferences for this population of digital mental health care seekers. Given that mental health providers have expressed concern that stepped care prioritizes economic benefits and discounts patient preferences [19], our study substantiates that stepped care may not only be a more scalable and equitable approach to mental health care, but it also has a more patient-centered model.

Our study also revealed that in this population of digital mental health care seekers, those who selected one-on-one care were more likely to have screened positive on the depression or anxiety screener, reported less well-being, endorsed greater functional impairment, and identified “my emotions” as the primary reason for seeking care. Thus, participants who preferred one-on-one care generally reported clinical severity factors and treatment focus areas, indicative of a greater need for higher levels of care. This finding suggests that in a stepped care delivery model, outpatient care seekers may have care modality preferences that are informed by their symptom severity, validating the need for a stepped care approach to mental health care. Such an approach is critical not only because it considers patient preferences for treatment, but it also allows for a more scalable model of mental health care. To elucidate this point, consider a world with no neighborhood pharmacies to accommodate nonlife-threatening care needs; individuals in need of health care would be left with no other choice but to seek out a top-of-license medical doctor for all medical ailments, regardless of symptom severity (eg, dry cough) and personal treatment preference (eg, trying over-the-counter medication first). Using a medical stepped care metaphor as a framework reveals that a mental health care landscape without a range of care options commensurate with varying degrees of symptom severity is antiquated.

For stepped care approaches toward mental health care to become viable, ethical, and patient-centered, it is essential to understand patient factors associated with different modality preferences. Although the largest number of participants (45%) expressed a preference for traditional one-on-one treatment, nearly half of the participants indicated a preference for self-directed care (24%) or being unsure of their preference (24%). Older age, being males, lower overall distress, and negative depression and anxiety screening results were significantly predictive of a preference for self-guided digital care. This suggests that many adults would prefer a self-guided digital approach to manage the challenges associated with subclinical psychological distress. This aligns with prior research that men and middle- and older-aged adults tend to seek less help for psychological distress [20,21]. Notably, our study revealed that 25% of the participants were unsure regarding their care modality preference. Participants unsure about their preference were more likely to be older than 45 years with lower overall distress, and negative depression and anxiety screening results. These results indicate that more psychoeducation about care modalities may be warranted for up to a quarter of care-seeking individuals to help patients self-determine their care preferences. Given that our sample included only individuals whose employers offered the Modern Health mental health benefit, it may be reasonable to assume that this is a particularly well-educated and well-resourced population. These results are likely to be more exaggerated in the general population that tends to have less mental health access and literacy. Future research should investigate the relationship between mental health literacy and prior experience with mental health care with perceived needs and preferences for different types of mental health care.

Technology-enabled mental health care delivery systems, though not commonly available to the general public, have the potential to approach psychological care in a way that is patient-centered and individualized to patient preferences and needs for treatment. Importantly, technology-enabled mental health platforms have the ability to ensure that patient care is collaborative between patients and providers and that patient values guide clinical decisions [22]. This study revealed that traditional one-on-one mental health care, which is frequently regarded as the “gold standard,” may not be preferred to the same extent across patients. When presented with the opportunity to choose, some patients prefer group care, self-directed treatment, or care options that are less rigidly structured (eg, meeting with a coach when needed instead of once a week/every week for months, meeting for 30-minute sessions, having check-ins once a month, and support via text messaging). A technology-enabled platform can customize care options based on preferences and perceived needs.

### Limitations

The sample of respondents in the current study is a relatively homogenous group of primarily younger adults having access to the Modern Health mental health benefit through their employer. Similarly, these respondents are likely to be generally healthier, better educated, and more financially stable than the general population, given their affiliation with the Modern Health employer-based benefit. As a result, these findings may not be generalizable to a more diverse sample. Future research should seek to confirm these results in a community-based sample with greater heterogeneity in the respondent characteristics. Another limitation of this study is that certain demographic variables were not collected (eg, race, ethnicity, and income); thus, our ability to completely characterize the sample was hindered. Future research can build upon this study to more comprehensively characterize the demographic variables associated with care modality preferences. Additionally, this study did not enquire participants about their previous experiences with mental health care, which is a factor likely to inform treatment modality preferences and mental health literacy. Future studies can seek to understand additional factors that influence patient preferences for mental health treatment modalities.

### Conclusions

This study revealed that care modality preferences for digital mental health treatment are variable based on demographic factors as well as clinical severity and area of focus indicators. This suggests that care modality preferences align with the innovations in mental health care delivery; one-on-one care with a provider is no longer the only or necessarily best option for many care seekers, as internet-delivered group and self-paced interventions have also shown strong clinical effectiveness for certain populations [14,23,24]. To provide efficient, scalable, and patient-centered mental health care, it is essential to continue understanding how best to funnel care seekers into different treatment modalities within a stepped care model. Our study revealed several key clinical and demographic factors that were associated with different care preferences, but future research should investigate how other important patient-level factors—including mental health literacy, race, ethnicity, and prior experience with the mental health care systems—impact care modality preferences and how aligning care recommendations with modality preferences affects care usage and treatment outcomes.

## Abbreviations

ANOVA: analysis of variance
CBT: cognitive behavioral therapy
GAD-2: Generalized Anxiety Disorder-2
mHealth: mobile health
OR: odds ratio
PHQ-2: Patient Health Questionnaire-2
WHO-5: World Health Organization-5 Well-being Index (WHO-5)
